# The FGFR4-G388R Polymorphism Promotes Mitochondrial STAT3 Serine Phosphorylation to Facilitate Pituitary Growth Hormone Cell Tumorigenesis

**DOI:** 10.1371/journal.pgen.1002400

**Published:** 2011-12-08

**Authors:** Toru Tateno, Sylvia L. Asa, Lei Zheng, Thomas Mayr, Axel Ullrich, Shereen Ezzat

**Affiliations:** 1 Ontario Cancer Institute, University Health Network, Toronto, Canada; 2 The Endocrine Oncology Site Group, Princess Margaret Hospital, Toronto, Canada; 3 Department of Medicine, University of Toronto, Toronto, Canada; 4 Department of Laboratory Medicine and Pathobiology, University of Toronto, Toronto, Canada; 5 Department of Molecular Biology, Max-Planck-Institute of Biochemistry, Martinsried, Germany; University of Michigan Medical School, United States of America

## Abstract

Pituitary tumors are common intracranial neoplasms, yet few germline abnormalities have been implicated in their pathogenesis. Here we show that a single nucleotide germline polymorphism (SNP) substituting an arginine (R) for glycine (G) in the FGFR4 transmembrane domain can alter pituitary cell growth and hormone production. Compared with FGFR4-G388 mammosomatotroph cells that support prolactin (PRL) production, FGFR4-R388 cells express predominantly growth hormone (GH). Growth promoting effects of FGFR4-R388 as evidenced by enhanced colony formation was ascribed to Src activation and mitochondrial serine phosphorylation of STAT3 (pS-STAT3). In contrast, diminished pY-STAT3 mediated by FGFR4-R388 relieved GH inhibition leading to hormone excess. Using a knock-in mouse model, we demonstrate the ability of FGFR4-R385 to promote GH pituitary tumorigenesis. In patients with acromegaly, pituitary tumor size correlated with hormone excess in the presence of the FGFR4-R388 but not the FGFR4-G388 allele. Our findings establish a new role for the FGFR4-G388R polymorphism in pituitary oncogenesis, providing a rationale for targeting Src and STAT3 in the personalized treatment of associated disorders.

## Author Summary

Several human cancers have been associated with increased growth hormone levels. Here we show that a frequent single nucleotide polymorphism (SNP) associated with increased cancer risk and progression also deregulates pituitary function. Through recruitment of a distinct STAT3 signaling cascade, this polymorphic receptor variant drives pituitary growth hormone cell survival and hormonal output. These findings provide an example of a potentially common genetic program shared between cancer and a hormone that promotes its progression.

## Introduction

Pituitary tumors occur in almost 20% of the population [1] and represent nearly 10% of surgically resected intracranial tumors [2]–[3]. They can cause significant health problems due to abnormal hormone production and invasion into surrounding brain structures [2]–[3]. However, the mechanisms underlying the development of sporadic pituitary tumors that rarely involve mutations of classical oncogenes or tumor suppressor genes remain to be clarified [2]–[3]. Indeed, the only consistent molecular event reported thus far is activating mutations of the G-protein coupled Gsα that occurs in a subset of somatotroph adenomas [4]–[5]. Germline genetic abnormalities associated with pituitary tumor pathogenesis include inactivating mutations of menin in patients with Multiple Endocrine Neoplasia type 1 [6]–[7], loss of function mutations of the aryl hydrocarbon receptor-interacting protein (AIP) tumor suppressor gene in patients with familial isolated pituitary adenomas [8], and activating mutations the Protein kinase A type I regulatory subunit PRKA [9] in patients with Carney complex, however these alterations have not been shown to mediate pituitary neoplastic growth in the more common sporadic neoplasms.

Evidence suggests that epigenetically controlled growth signals implicated in pituitary development may be relevant to the tumorigenic processes in this gland [10]–[11]. Of note members of the fibroblast growth factor (FGF) and FGF receptor families have been proposed as candidate effectors, given their recognized importance in pituitary organogenesis [12]–[13]. FGF signaling is critical in pituitary development. Deletion of FGF10 or its receptor, the FGFR2 IIIb isoform, leads to failure of pituitary development [13]. Mid-gestational expression of a soluble dominant-negative FGFR results in severe pituitary dysgenesis [14]. FGF ligands are over-expressed in pituitary tumors. FGF-2, originally described in bovine pituitary folliculostellate cells, regulates multiple pituitary hormones and is over-expressed by human pituitary adenomas tumors [15].

We identified altered FGFR4 expression in pituitary tumors [16] due to expression of an N-terminally deleted isoform, pituitary tumor-derived FGFR4 (ptd-FGFR4) [17] generated by alternative transcription initiation from a cryptic promoter [18]–[19]. Prototypic FGFR4 (FGFR4-G388) is a 110 kD membrane-anchored protein expressed in several endocrine cells including the normal pituitary. In contrast, ptd-FGFR4 is a cytoplasmic protein expressed in pituitary tumors. The invasive tumorigenic potential of ptd-FGFR4, but not full length FGFR4, was demonstrated by targeted pituitary expression in transgenic mice [17]. The basis for the contrasting functions between these FGFR4 isoforms relates to their differential ability to associate with neural cell adhesion molecule (NCAM) and engage N-cadherin [20].

These studies were all carried out with the prototypic receptor prior to the identification of a single nucleotide polymorphism (SNP) that alters the coding region of the transmembrane domain. This germ-line polymorphism substitutes a glycine with an arginine at codon 388 of FGFR4, resulting in a charged amino acid in the highly conserved and normally hydrophobic transmembrane region of the receptor [21]. This FGFR4-R388 allele has been linked with advanced [21] and treatment-resistant breast cancer [22], prostate cancer [23], sarcomas [24], and head and neck carcinomas [25]. The mechanisms underlying FGFR4-R388 actions remain unclear. In this report we identify distinct signaling and hormone regulatory properties that distinguish FGFR4-R388 from the prototypic FGFR4-G388 form. The data unmask important patho-physiologic consequences of this common SNP with therapeutic implications for related diseases.

## Results

### The FGFR4-R388 polymorphic allele deregulates pituitary hormone production and cell growth

**Figure 1:**
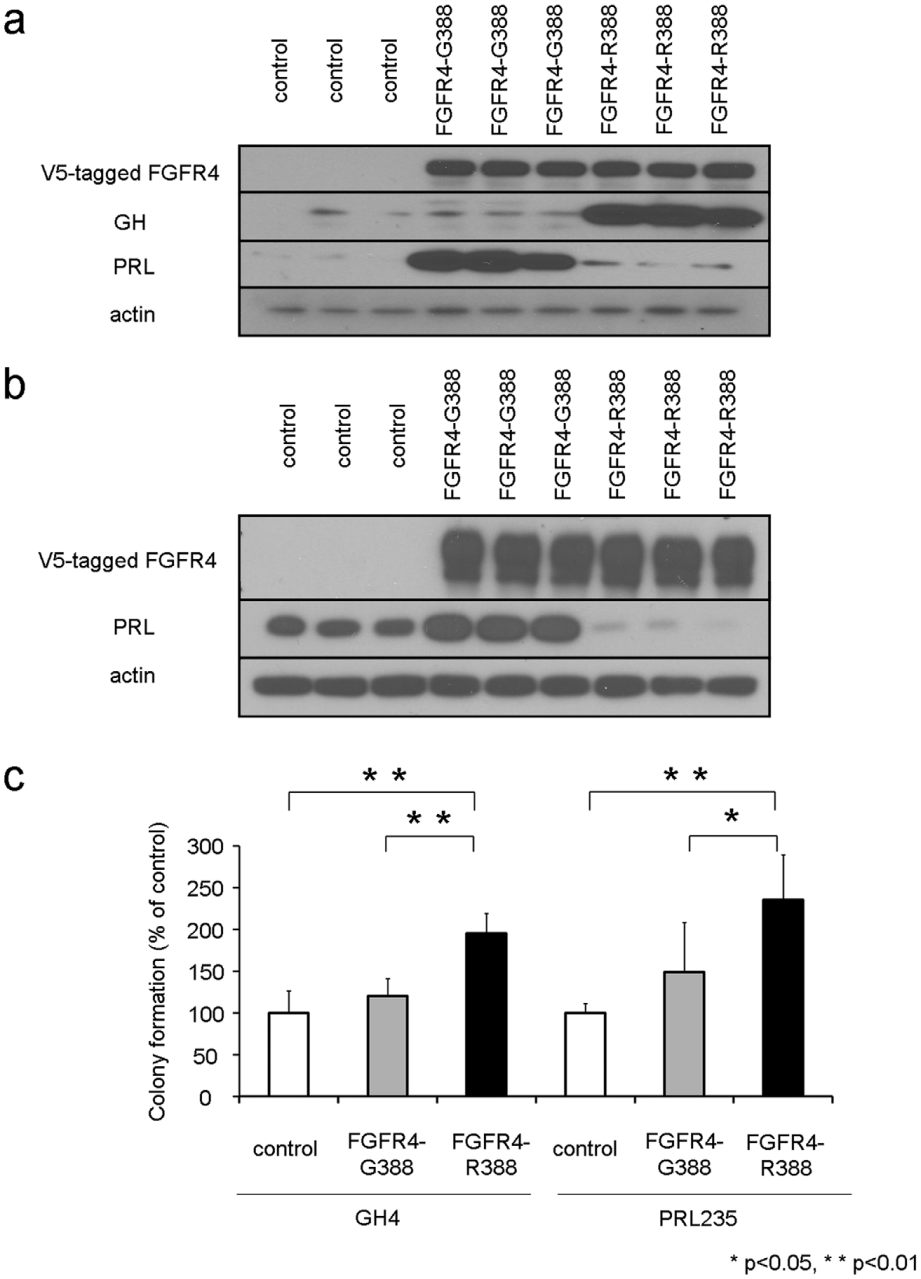
The FGFR4-R388 polymorphism deregulates pituitary hormone production and cell growth. (a) FGFR4, prolactin (PRL), and growth hormone (GH) protein expression were examined in pituitary GH4 mammosomatotroph cells stably expressing empty vector (control), the prototypic receptor (FGFR4-G388), or the human polymorphic FGFR4-R388 variant. FGFR4-G388 enhances PRL production whereas FGFR4-R388 increases GH expression. (b) FGFR4 and PRL protein expression were examined in pituitary PRL235 lactotroph cells stably expressing empty vector (control), the prototypic receptor (FGFR4-G388), or the human polymorphic FGFR4-R388 variant. FGFR4-G388 enhances PRL whereas FGFR4-R388 diminishes PRL expression. (c) GH4 cells or PRL235 cells expressing empty vector, FGFR4-G388, or FGFR4-R388 were plated in soft agar as described under materials and methods. Shown is the change in the number of colonies (mean ± SD obtained from four independent experiments). Statistically significant (p<0.05) increased colony numbers were noted in cells expressing FGFR4-R388 as indicated.

To determine if the FGFR4 polymorphic isoforms possess distinct functional properties in hormone-producing pituitary cells, we compared the effects of FGFR4-G388 and FGFR4-R388 on pituitary hormone production in rat GH4 mammosomatotroph cells that co-express prolactin (PRL) and growth hormone (GH) and in PRL235 cells that express PRL only. These GH4 and PRL235 cells express endogenous FGFR4 (Figure S1) and are homozygous for FGFR4-G385, the rodent equivalent of the human 388 site. Expression of human FGFR4-G388 or FGFR4-R388 to comparable levels shows that FGFR4-G388 enhances PRL and suppresses GH expression whereas FGFR4-R388 increases GH production with a reciprocal effect on PRL (Figure 1a, 1b).

To determine the effects of these FGFR4 isoforms on cell growth, stably transfected cells were plated in soft agar and examined for colony formation. GH4 cells expressing FGFR4-R388 were more efficient at forming colonies in soft agar compared with their FGFR4-G388 counterparts (Figure 1c; left). Enhanced colony formation resulting from FGFR4-R388 compared to FGFR4-G388 was also noted in PRL235 pituitary cells (Figure 1c; right).

### The FGFR4-R388 polymorphic allele promotes Src-dependent responses in pituitary cells

**Figure 2:**
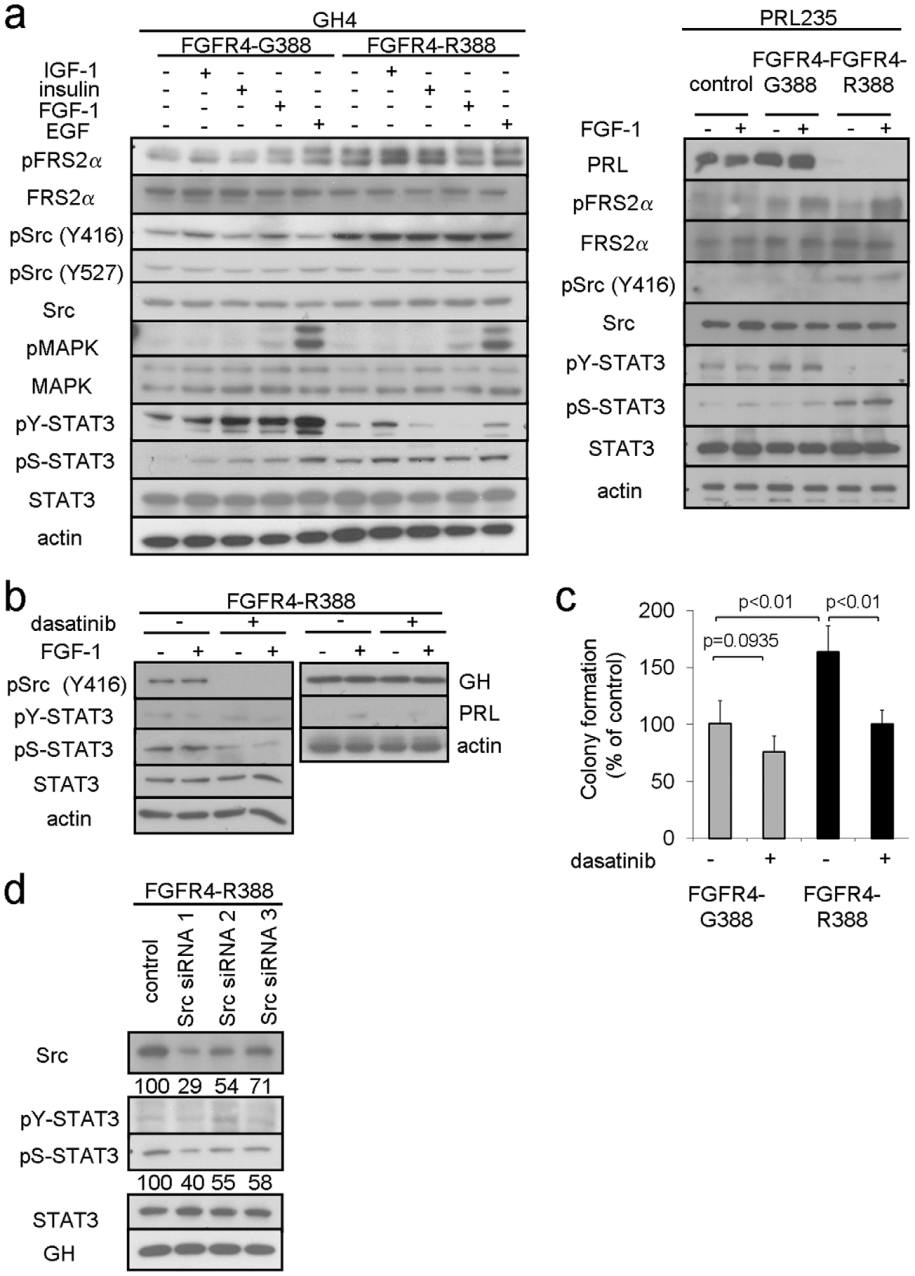
FGFR4-R388 promotes Src and STAT3 signaling in pituitary tumor cells. (a) GH4 cells (left) and PRL235 cells (right) expressing FGFR4-G388 or FGFR4-R388 were treated with the indicated panel of ligands under serum-free defined conditions. Equal amounts of cell lysates were resolved by SDS-PAGE and analyzed by immunoblotting with the indicated antibodies. Results are representative of three independent experiments. Compared to FGFR4-G388 cells which show ligand-dependent stimulation of FRS2α, FGFR4-R388 cells display enhanced FRS2α phosphorylation. Src phosphorylation at Y416 is also appreciably higher in cells expressing FGFR4-R388 compared to those expressing FGFR4-G388 while Src phosphorylation at Y527 is unchanged. While FGFR4-G388 supports ligand-induced tyrosyl phosphorylation of STAT3 at Y705, this effect is not shared with FGFR4-R388, which instead results in sustained STAT3 phosphorylation at S727. (b) GH4 cells expressing FGFR4-R388 were treated with FGF1 under serum-free defined conditions in the presence or absence of the Src inhibitor dasatinib as detailed under Materials and Methods. Dasatinib reduces Src phosphorylation and results in diminished pS-STAT3. Pituitary GH and PRL hormone levels, however, are unaffected by this Src inhibition. (c) GH4 cells expressing FGFR4-G388 or FGFR4-R388 were grown in soft agar in the absence or presence of dasatinib. Shown are the colony numbers expressed as the percent of control (mean ± SD of measurements obtained from four independent experiments). Dasatinib significantly inhibit the enhanced growth of cells expressing FGFR4-R388. (d) GH4 pituitary cells expressing FGFR4-R388 were transfected with siRNA targeting Src or a scrambled control and examined by Western blotting. Densitometric values of knockdown relative to controls are shown immediately below.

The FGFR4-R388 substitution does not alter receptor kinase activity [21] and (data not shown). Thus, to examine signaling differences induced by the two FGFR4 isoforms in pituitary cells, we compared the ability of FGF to promote phosphorylation of the immediate FGFR substrate FRS2α. In contrast to FGFR4-G388 which showed ligand-dependent stimulation of this docking protein, FGFR4-R388 cells displayed enhanced FRS2α phosphorylation (Figure 2a). Src phosphorylation at Y416 was also appreciably higher in cells expressing FGFR4-R388 compared to those expressing FGFR4-G388 while Src phosphorylation at Y527 remained unchanged.

To examine the functional significance of this finding, we compared the ability of the Src inhibitor, dasatinib, to impede pituitary tumor cell growth and hormone production. Dasatinib effectively diminished Src phosphorylation (Figure 2b) and significantly inhibited colony formation in soft agar of cells expressing FGFR4-R388 (Figure 2c). By comparison, the less efficient colony forming FGFR4-G388 cells were relatively insensitive to the Src inhibitor (Figure 2c).

In contrast to the impact on cell growth, pharmacologic Src inhibition did not alter GH or PRL hormone expression (Figure 2b). Additionally, siRNA-mediated Src down-regulation did not significantly affect GH (Figure 2d) or PRL levels (data not shown). These findings suggested that while Src may play a role in driving FGFR4-R388-mediated cell growth, Src signaling may not be intimately coupled with pituitary hormone regulation in these cells.

### FGFR4-R388 promotes STAT3 serine, but not tyrosyl, phosphorylation in pituitary cells

STAT activation is implicated in mediating the effects of FGFR3 mutations associated with thanatophoric dysplasia [26]. We, therefore, examined the ability of the two FGFR4 isoforms to activate STAT signaling. Figure 2a depicts the differential impact of the FGFR4 isoforms on their ability to phosphorylate STAT3. While FGFR4-G388 supported ligand-induced tyrosyl phosphorylation of STAT3, this effect was not shared with FGFR4-R388, which instead resulted in sustained STAT3 serine phosphorylation at S727 (Figure 2a). STAT1 and STAT5 modifications were not affected by either FGFR4 isoform in GH4 or PRL235 cells (data not shown).

**Figure 3:**
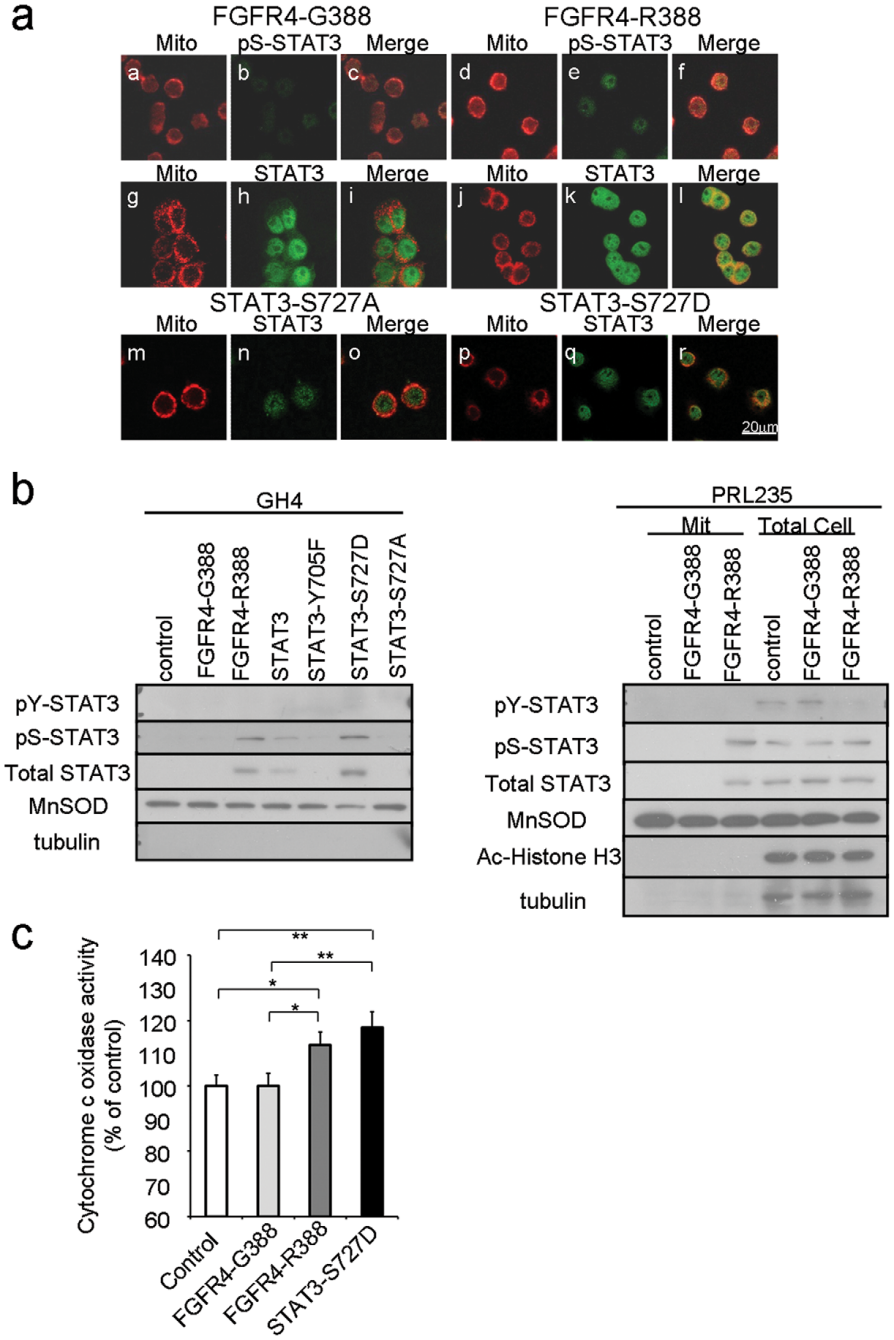
FGFR4-R388 relies on STAT3 serine phosphorylation to induce mitochondrial Cytochrome c activity and pituitary tumor cell growth. GH4 pituitary cells stably expressing FGFR4-G388 (a–c, g–i), FGFR4-R388 (d–f, j–l), STAT3-S727A (m–o), or STAT3-S727D (p–r) were labeled with MitoTracker RED CMX Ros to visualize mitochondria under serum-free defined conditions, fixed, and stained with anti-phospho-serine STAT3 or anti-STAT3 followed by Alexa 488-conjugated secondary antibody. (b) Equal amounts of fractionated proteins from cells expressing FGFR4-G388 or FGFR4-R388 were resolved by SDS-PAGE and immunoblotted for detection for pS-STAT3 and the following markers: Ac-Histone H3 (nuclear), MnSOD (mitochondrial), or tubulin (cytoplasmic) as indicated. Results are representative of three separate experiments. (c) Cytochrome c oxidase was measured as an index of mitochondrial metabolic activity. Shown are the mean values from 3 independent experiments. Statistically significant differences are depicted as *p<0.05 or **p<0.01.

In contrast to the nuclear residence of pY-STAT3, pS-STAT3 translocates to the mitochondria where it has been implicated in cellular metabolism [27]. Thus, we performed immunofluorescence to localize pS-STAT3. Figure 3a identifies the mitochondrial residence of pS-STAT3 in FGFR4-R388; FGFR4-G388 cells are almost negative (upper panels). As controls, we expressed a constitutively active serine form of STAT3 (STAT3-S727D) in GH4 cells, and this also co-localized to the mitochondria (Figure 3a). In contrast, an inactive serine form of STAT3 (STAT3-S727A) failed to show a mitochondrial signal (Figure 3a). Subcellular fractionation followed by western blotting supported these findings with prominent mitochondrial expression of STAT3 and pS-STAT3 in FGFR4-R388 and in STAT3-S727D control but not in FGFR4-G388 cells in both GH4 and PRL235 cells (Figure 3b).

To examine the impact of pS-STAT3 on mitochondrial function, we measured Cytochrome C oxidase activity in pituitary cells expressing the different FGFR4 isoforms (Figure 3c). FGFR4-R388 cells which displayed higher pS-STAT3 levels also demonstrated higher Cytochrome C oxidase activity than FGFR4-G388 cells. In addition, lactate dehydrogenase (LDH) levels in FGFR4-R388 cell lysates were higher (2612 IU/ml) than those of the FGFR4-G388 (1197 IU/ml; n = 3).

To examine the functional impact of distinct STAT3 modifications on pituitary cells we compared growth in soft agar of cells expressing various STAT3 expression vectors (Figure S2). Of the STAT3 modifications, the active serine (STAT3-S727D) form displayed the greatest positive impact on colony formation (Figure S2).

To determine whether Src and STAT3 serine phosphorylation were inter-dependent, we examined the impact of pharmacologic Src inhibition. To this end, dasatinib-mediated inhibition of Src phosphorylation also reduced pS-STAT3 in FGFR4-R388 GH4 cells (Figure 2b). Moreover, siRNA-mediated Src down-regulation also resulted in diminished pS-STAT3 levels in these cells (Figure 2d). Additionally, treatment with the protein kinase C inhibitor G06983 reduced pS-STAT3 whereas the protein kinase A inhibitor H89 had no effect (data not shown).

### Dysregulated STAT3 signaling by FGFR4 variants interrupts growth hormone feedback

**Figure 4:**
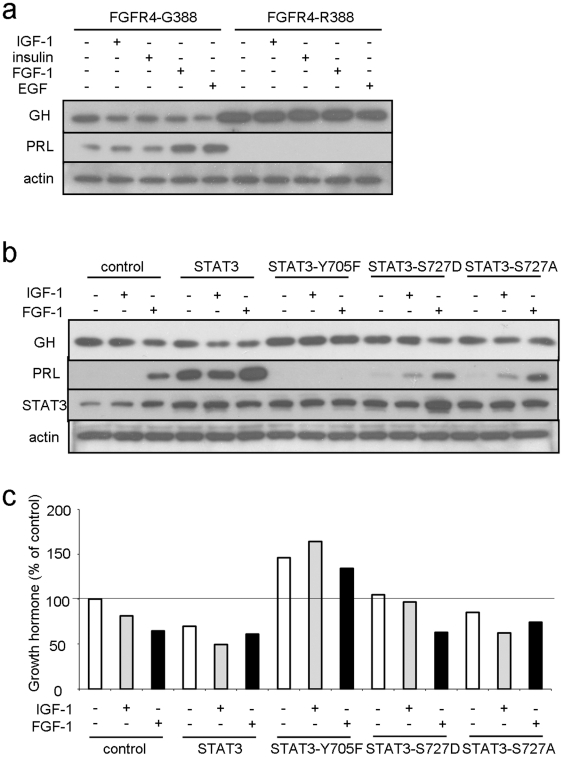
FGFR4-G388, but not FGFR4-R388, signals through pY-STAT3 to regulate pituitary hormone gene expression. (a) Pooled GH4 cells expressing FGFR4-G388 or FGFR4-R388 were treated with the indicated ligands for 24 hrs under serum-free defined conditions. Equal amounts of cell lysates were resolved by SDS-PAGE and immunoblotting detection for PRL, GH, or actin as indicated. Results are representative of three separate experiments. (b) GH4 cells expressing empty vector (control), wild-type (STAT3), dominant negative (STAT3-Y705F), constitutively active STAT3-S727D, or inactive STAT3-S727A were treated with the indicated ligands for 24 hrs under serum-free defined conditions. Equal amounts of cell lysates were resolved by SDS-PAGE and immunoblotting detection for PRL, GH, STAT3, or actin as indicated. (c) Densitometric ratios from three separate experiments are shown immediately below.

To determine whether differential STAT3 responses were responsible for altered hormone gene expression, we compared the hormonal responses of FGFR4-G388 and FGFR4-R388 cells to a panel of growth factors. FGFR4-G388 cells with intact pY-STAT3 responses exhibited the expected GH inhibition in the presence of IGF-1 and related growth factors and the expected PRL increase in response to growth factors (Figure 4a). In contrast, and consistent with their attenuated pY-STAT3 responses, FGFR4-R388-expressing cells failed to suppress GH or mount a PRL response (Figure 4a).

In complementary experiments we down-regulated STAT3 in FGFR4-G388 cells. This forced STAT3 reduction resulted in increased GH and reduced PRL (Figure S3). Conversely, forced expression of STAT3 induced PRL and diminished GH expression (Figure 4b, 4c). Moreover, introduction of the dominant negative STAT3-Y705F diminished IGF-1 inhibition of GH (Figure 4b, 4c). The STAT3-Y705F mutant also failed to stimulate PRL expression further underscoring the requirement for pY-STAT3 in mediating PRL induction. In contrast to the impact of pY-STAT3, introduction of the constitutively active serine STAT3-S727D or the serine inactive STAT3-S727A did not alter GH or PRL responses (Figure 4b, 4c).

Taken together, these findings suggest that pY-STAT3, but not pS-STAT3, plays a more important role in pituitary GH and PRL regulation.

### The FGFR4 polymorphism facilitates pituitary tumor formation *in vivo*

**Figure 5:**
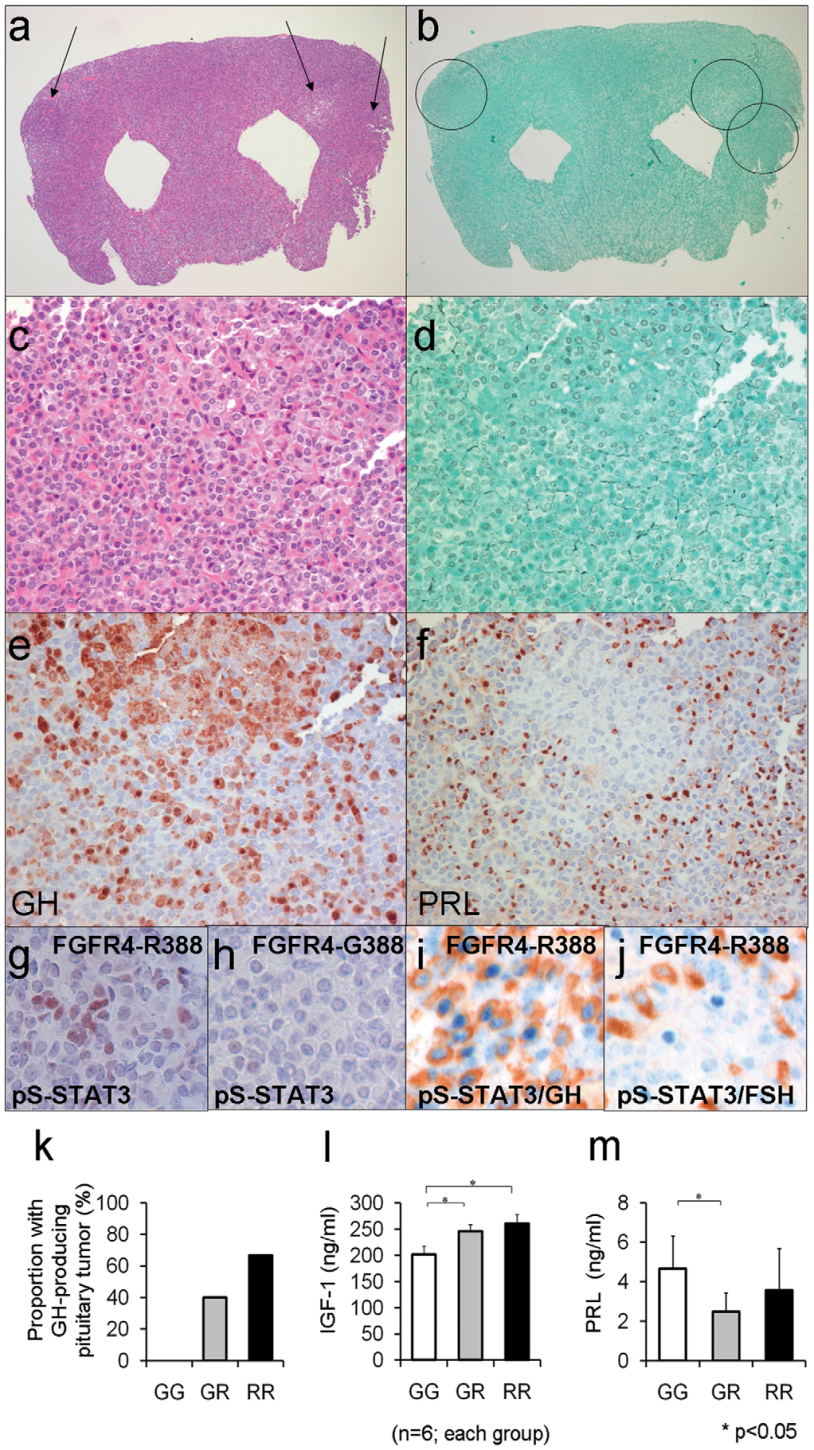
Mouse knock-in of the FGFR4 SNP facilitates pituitary GH cell tumorigenesis. (a) The pituitary glands of Fgfr4-R385 knock-in mice reveals multiple adenomas (arrows) (H&E stain; magnification ×10). (b) The Gordon Sweet silver stain confirms loss of acinar structures (magnification, ×15). (c) The polygonal tumor cells form cords and nests (H&E stain; magnification ×150). (d) The reticulin stain shows the nodules exhibit total breakdown of the reticulin fiber network, diagnostic of adenoma (Gordon Sweet silver stain; magnification, ×150). (e) GH immunoreactivity is identified in somaototroph cells and strongly in adenoma cells (Avidin-biotin-peroxidase complex technique; magnification, ×200). (f) In the pituitary of the knock-in mouse, PRL immunoreactivity is identified in mammosomatotroph cells but not in adenoma cells (Avidin-biotin-peroxidase complex technique; magnification, ×200). (g) Phospho-serine (pS727-STAT3) immunoreactivity is identified in pituitary cells of Fgfr4-R385^(+/+)^ mice but not (h) FGFR4-G385 (wild type) mice. (i) pS727-STAT3 immunoreactivity is co-localized in GH-expressing cells of Fgfr4-R385^(+/+)^ mice, but not (j) gonadotroph cells of the same animals. (k) The frequency of pituitary tumor development in mice for Fgfr4-385 (G/G), heterozygote (G/R), or homozygote (R/R) is shown. (l) Circulating IGF-1 levels as an integrated measure of pituitary GH output or prolactin (PRL) (m) were compared in mice of the corresponding FGFR4 genetic background. Shown are the mean ±SEM of 6 mice in each group where statistically significant differences (p<0.05) were noted.

To determine if the observed cellular actions of FGFR4 polymorphism translate into biologically relevant actions on pituitary cell growth and function, we examined mice with knock-in (KI) of the mouse homologue of the polymorphism, Fgfr4-R385. Importantly, introduction of this SNP does not alter Fgfr4 expression levels [28] and (Figure S1). Systematic examination of the pituitary glands from mice carrying the Fgfr4-R385 allele at different ages identified the presence of pituitary tumors by 12 months of age. This revealed increased cellularity with loss of the reticulin network representing the hallmark of true neoplasia in this gland (Figure 5a–5d). Importantly, unlike the more common prolactinomas which are seen sporadically in aging mice [29], hormone staining identified GH (Figure 5e) but not PRL (Figure 5f) production by these tumors. Further, we examined pS-STAT3 in pituitary tissue from the knock-in mouse model. Fgfr4-R385 KI mice displayed strong immunoreactivity for pS-STAT3 (Figure 5g) which was not noted in control Fgfr4-G385 mice (Figure 5h). Double staining localized this pS-STAT3 in GH- immunoreactive somatotrophs (Figure 5i) but not in other cell types such as FSH-immunoreactive gonadotrophs (Figure 5j).

The frequency of these pituitary tumors and their morphologic phenotypes according to Fgfr4 genotype are summarized in Figure 5k. No GH-containing pituitary tumors were detected in control littermates (Figure 5k).

To corroborate the pituitary phenotypic abnormalities we compared circulating levels of the GH target growth factor IGF-1. Shown in Figure 5l is the positive impact of the Fgfr4-R385 allele on circulating IGF-1 levels. In contrast, and consistent with the *in vitro* data, mice carrying the Fgfr4-R385 allele did not demonstrate high PRL levels, and instead showed a tendency to lower concentrations compared to their Fgfr4-G385 littermates (Figure 5m).

### The FGFR4-R388 allele correlates with GH pituitary tumor phenotype in humans

**Figure 6:**
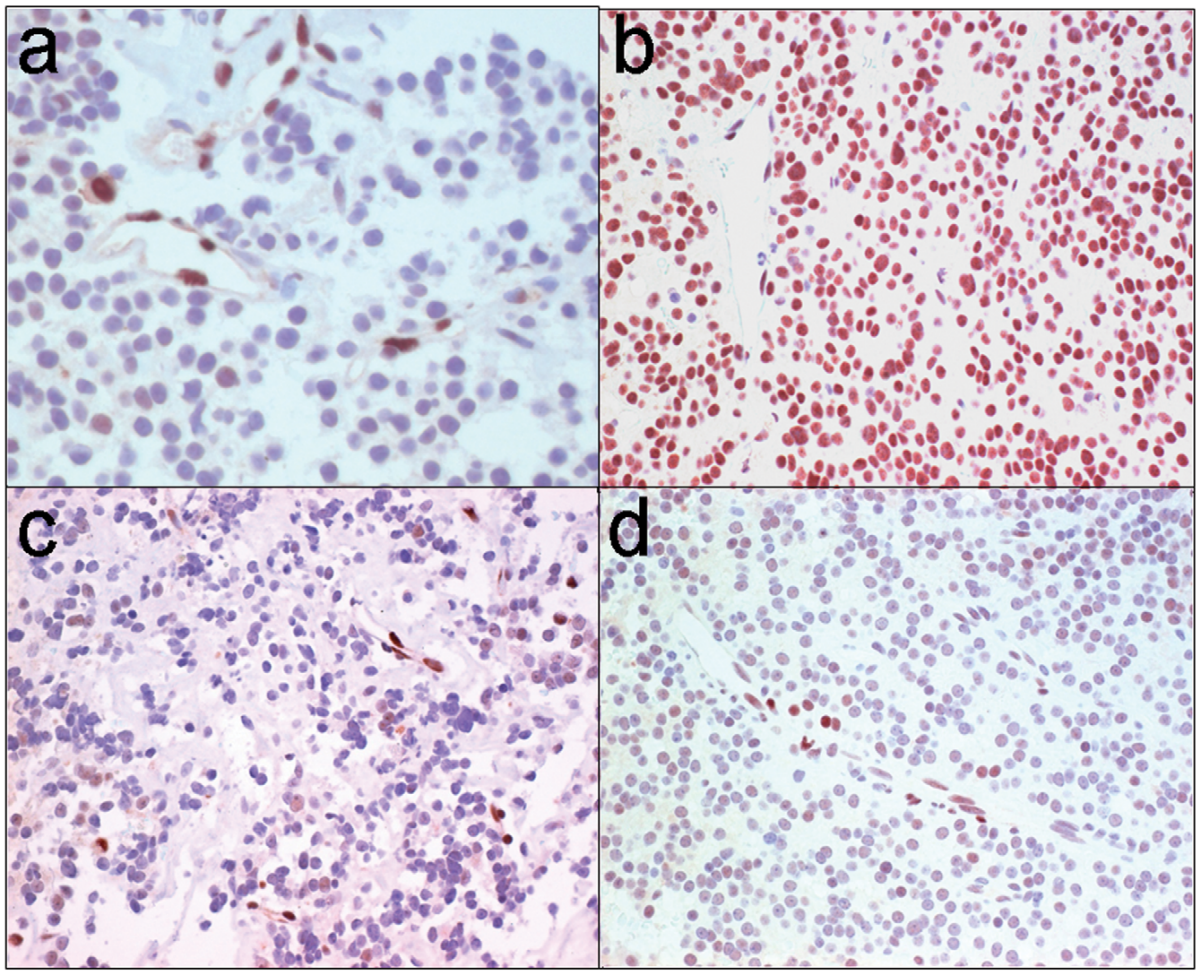
STAT3 serine phosphorylation in human GH pituitary tumors. (a) Immunohistochemistry for phospho-serine (pS727-STAT3) in the normal adenohypophysis reveals reactivity in vascular endothelium providing an internal positive control; there is focal faint staining in scattered adenohypophysial cells and stroma is largely negative. (b) All but one of 8 somatotroph (GH) adenomas exhibited strong positivity whereas (c) all lactotroph (PRL) adenomas (n = 4) were negative or showed focal weak positivity. (d) Ten of 13 non-functional adenomas (6 gonadotrophs, 7 null cell) were also either negative or weakly positive.

Given the ability of FGFR4-R388 to facilitate pituitary tumorigenesis and GH production, we sought to identify evidence linking these two processes in human disease. We first examined STAT3 serine phosphorylation in human pituitary tissue. In the normal gland, immunohistochemistry for pS-STAT3 revealed strong reactivity in vascular endothelium (Figure 6a), providing an internal positive control; adenohypophysial cells and stroma were largely negative or showed only faint staining. All but one of 8 somatotroph adenomas exhibited strong positivity (Figure 6b) whereas lactotroph adenomas (n = 4) were negative or showed focal weak positivity (Figure 6c). Four of 6 gonadotroph adenomas and all but one of 7 null cell adenomas were also either negative or weakly positive (Figure 6d).

We next compared GH levels and pituitary tumor size in 64 patients with pituitary tumors and acromegaly based on their FGFR4 genotypic status. This examination identified a positive correlation between circulating GH levels (r = 0.622, p = 0.006) and pituitary tumor size in patients (n = 30) harboring an FGFR4-R388 allele. In contrast, patients homozygous for FGFR4-G388 (n = 34) showed no relationship (r = 0.23; p = 0.468) between GH levels and pituitary tumor size. Additionally, there was no relationship between FGFR4 genotype and tumor size in non-functional gonadotroph pituitary tumors (n = 22) or lactotroph adenomas (n = 13). These data support a selective link between the FGFR4-R388 allele, GH hormone production, and clinical pituitary somatotroph tumor formation.

## Discussion

The FGFR4-R388 SNP is known to promote breast cancer cell motility and invasiveness [21]. It has also been associated with accelerated cancer progression and treatment resistance [21]–[25]. However, the mechanisms underlying these actions remain unclear. We show here that FGFR4-R388 significantly alters pituitary function. Compared with the prototypic form of the receptor (FGFR4-G388), the polymorphic FGFR4-R388 variant supports distinct signaling to deregulate pituitary growth hormone production and cell growth *in vitro* and *in vivo*. In the mouse model we report, Fgfr4 expression levels are not altered [28], providing relevance to the human situation. Unlike the common sporadic pituitary lactotroph adenomas in rodents or the intermediate lobe corticomelanotroph pituitary tumors associated with several mouse models of cancer [29], the Fgfr4 SNP knock-in mice develop GH-producing pituitary tumors. The resulting GH/IGF-1 excess in these animals is potentially important in the enhanced breast cancer progression associated with this model [28].

FGFR signaling relies heavily on recruitment of the immediate substrate FRS2α through tyrosyl phosphorylation [30]. Using hormone-producing pituitary cells we show that compared to FGFR4-G388, FGFR4-R388 is associated with enhanced phosphorylation of FRS2α but not with the anticipated downstream MAPK activation. Instead, FGFR4-R388 signaling is accompanied by enhanced Src and STAT3 activation in pituitary cells. Consistent with this feature, pharmacologic Src inhibition results in greater growth inhibition by pituitary cells expressing FGFR4-R388. Neither pharmacologic inhibition nor Src knockdown, however, could alter the GH excess associated with FGFR4-R388. Instead, the FGFR4 SNP variant relies heavily on serine (S727) but not tyrosyl (Y705) phosphorylation of STAT3. These findings suggested that while Src may play a role in promoting cell growth, the observed hormone dysregulation was not intimately coupled with this putative oncogene.

The current study implicates multiple consequences of altered STAT3 modifications in the control of pituitary hormonal balance. As anticipated, FGFR4-G388 supports FGF-induced FRS2 phosphorylation to promote pY705-STAT3 activation. In turn, pY705-STAT3 induces PRL, as has been shown previously [31]. Conversely, the attenuated pY-STAT3 response, likely the result of pS-STAT3 [32], associated with the FGFR4-R388 SNP relieves GH from inhibition leading to higher expression of this hormone. STAT3 is a well-recognized mediator of cytokine signaling, and is known to regulate GH produced by the pituitary gland [27]. Interestingly, STAT5 which is also implicated in GH regulation [33] is not affected by this FGFR4 SNP in pituitary cells.

Pituitary auto-feedback mechanisms are candidate pathways whose interruption has become increasingly well-appreciated [2]–[3]. PRL receptor knockout mice develop pituitary lactotroph tumors [34]. Similarly, a somatic pituitary tumor-associated mutation in the extracellular domain of the GH receptor (GHR) disrupts N-terminal glycosylation of the receptor, thereby impairing GHR trafficking to the membrane, limiting ligand binding, and disrupting auto-feedback inhibition through diminished STAT activation [35]–[36]. Insulin and IGF-1 are growth factors that are known to exert negative-feedback at the level of the pituitary to inhibit GH [37]. In our study, insulin/IGF-1 efficiently activated pY-STAT3 to inhibit GH expression. In contrast, FGFR4-R388 failed to activate pY-STAT3 following ligand stimulation. The importance of diminished pY-STAT3 in mediating increased GH expression was further gleaned from knockdown of this STAT. STAT3 down-regulation or introduction of dominant negative STAT3-Y705F resulted in augmented GH production. Taken together, these data support the importance of STAT3 in the feedback inhibition control of GH regulation. In the bi-hormonal mammosomatotroph cell line examined, this altered signaling had the reverse effect on PRL. STAT3 down-regulation reduced PRL, whereas forced expression of STAT3 increased PRL and reduced GH expression by facilitating IGF-1 action. It is noteworthy that mice lacking pS-STAT3 have reduced IGF-1 levels [38] providing a complementary model to the increased IGF-1 noted in our FGFR4-R385 KI mice with pituitary somatotroph gain of pS-STAT3.

In contrast to the impact of FGFR4-G388 on pY-STAT3, FGFR4-R388 was associated with serine STAT3 phosphorylation (S727-STAT3). STAT3 is generally regarded as a requirement for Src-mediated cell transformation as shown in many carcinomas [39]. Traditionally, STAT3 oncogenic functions have been regarded to rely on pY-STAT3 and its nuclear translocation. However, more recently the positive impact of pS727-STAT3 on cell transformation has emerged. Unlike the tyrosyl modification, serine phosphorylated STAT3 has been also been described in the mitochondria [40] and negatively modulates tyrosyl phosphorylation [32]. Mitochondrial pS-STAT3 as shown in this study has been implicated in augmented electron transport complex and ATP synthase activity to yield higher lactate dehydrogenase [27], a critical metabolic requirement for transformed cells.

Previous studies have shown that pharmacologic inhibition of wild-type FGFR4 was not effective in arresting pituitary tumor xenografts [41]. Given our newly recognized FGFR4-R388 ability to preferentially activate Src in pituitary cells, we set out to re-examine the potential role of pharmacologic interruption on pituitary tumor-associated parameters. Using the Src inhibitor dasatinib [42], we demonstrate the ability of this agent to inhibit colony formation by FGFR4-R388 pituitary tumor cells. Given the recognized oncogenic actions of Src [43], and specifically in pituitary tumorigenesis as shown here, our data provide new insights into how this kinase might be an attractive therapeutic target in patients harboring the FGFR4-R388 SNP. It is equally plausible that inhibitors of STAT3 and those targeting oxidative phosphorylation may be of potential value in modulating pituitary tumors for therapeutic purposes.

In summary, we show that the heritable FGFR4-R388 allele yields a receptor variant that signals in a distinct manner from its prototypic FGFR4-G388 form in pituitary cells. Through its preferential ability to activate Src and pS-STAT3, FGFR4-R388 facilitates pituitary cell transformation. Further, the diminished ability to respond through pY-STAT3 results in attenuated negative feedback inhibition to augment pituitary GH expression. Given the recognized impact of FGFR4-R388 [21]–[25] and of the GH/IGF-I axis on cancer progression [44], the current findings identify the common FGFR4 polymorphism as an endocrine signal participating in these processes. It also highlights Src and STAT3 as potential targets for the treatment of patients with growth disorders in the context of the FGFR4 transmembrane polymorphism.

## Materials and Methods

### Cell lines and cultures

As there are no human-derived hormone-producing pituitary cell lines, we used rat pituitary GH4 mammosomatotroph cells which were propagated in Ham F10 medium 12.5% horse and 2.5% fetal bovine serum (FBS; Sigma, Oakville, ON), 2 mM glutamine, 100 IU/ml penicillin, and 100 µg/ml streptomycin (37°C, 95% humidity, 5% CO2 atmosphere incubation). Rat pituitary PRL235 lactotroph cells were propagated in DMEM 10% FBS, 2 mM glutamine, 100 IU/ml penicillin and 100 g/ml streptomycin.

### Plasmids and transfection

Plasmids encoding human prototypic FGFR4 (G388) or the polymorphic form FGFR4-R388 were generated and stably transfected into GH4 and PRL235 cells as previously described [17]. Construct fidelity was confirmed by DNA sequencing after introduction into pcDNA3.1. STAT3 expression vector was kindly provided by M. Minden (University of Toronto), dominant negative STAT3-Y705F, inactive STAT3-S727A, or constitutively active STAT3-S727D were kindly provided by J. Chen (University of Illinois) [42]. Cells were transfected using Lipofectamine 2000 (Life Technologies, Rockville, MD) according to the manufacturer's instructions. Stable clones were selected using neomycin (G418) at a concentration of 0.7 µg/ml. A minimum of 3 clones of each isoform were pooled for further analyses in each of the cell types examined.

### siRNA knockdown

Oligonucleotides complementary to the gene of interest were synthesized by Ambion and introduced by transfection using lipofectamine 2000. Scrambled sequences of equal length were used as controls.

### Cell treatments

Ligand stimulations were performed on cells grown in 100 mm plate (4×10^6^ cells/plate), pre-incubated as indicated for 1 hr or 24 hrs in serum-free defined medium (3 µg/ml putresine, 10-6 M hydrocortisone, 10-11 M tri-iodothyronine T3, and 0.375% albumin bovine factor V), without or with dasatinib (Sequoia Research Products Ltd, Pangourne, UK, 100 nM), H-89 (Calbiochem, San Diego, CA, 1 µM), or G06983 (Sigma, 1 nM). Treatments with IGF-1 (Sigma, 13 nM), insulin (Eli Lilly, 600 nM), FGF-1 (Sigma, 25 ng/ml) with heparin (10 U/ml), and EGF (R & D systems, 25 ng/ml) were based on earlier dose and time course studies ranging from 5 min up to 24 hrs.

### Growth in soft agar

Twenty five hundred cells were plated in 35 mm dishes as a single cell suspension in 0.3% agar in Ham F10 medium supplemented with 15% horse serum and 10% CS over under-layer of 0.5% agar prepared in Ham F10 as above. For Src inhibition, cells were incubated with the pharmacologic inhibitor dasatinib at concentrations ranging from 10^−6^ to 10^−4^ M. Colony formation was monitored daily with a light microscope and colonies photographed 4 weeks later as previously described [43].

### Mitochondrial isolation and cytochrome c oxidase activity

Cells were lysed by mechanical homogenization for mitochondrial extraction using Qproteome Mitochondria isolation kit (Qiagen). Isolated fractions were analyzed by Western blotting to detect the MnSOD mitochondrial marker. Effective exclusion of contaminating cytoplasmic or nuclear proteins was confirmed by detection of tubulin and acetylated histone 3 respectively. Cytochrome C oxidase activity was used as a measure of electron transport chain activity according to the manufacturer's (Sigma) instructions.

### Western blotting and antibodies

Cells were lysed in lysis buffer (0.5% sodium deoxycholate, 0.1% sodium dodecyl sulfate, 1% Nonidet P-40 and 1× PBS) containing proteinase inhibitors (100 µg/ml phenylmethylsulfonyl fluoride (PMSF), 13.8 µg/ml aprotinin (Sigma), and 1 mM sodium orthovanadate (Sigma). Total cell lysates were incubated on ice for 30 mins, followed by micro-centrifugation at 10,000 g for 10 min at 4°C. Protein concentrations of the supernatants were determined by Bio-Rad method. Equal amounts of protein (50 µg) were mixed with 5× SDS sample buffer, boiled for 5 mins and separated by 8, 10, or 12% sodium dodecyl sulfate (SDS)-polyacrylamide gel electrophoresis, and transferred onto PVDF membranes (0.45 µm, Millipore, US). Intracellular and secreted hormones were determined using the following antibodies: polyclonal antisera to PRL or GH [donated by the National Hormone and Pituitary Program (NHPP), National Institute of Diabetes and Digestive and Human Development, Bethesdsa, MD] applied at dilutions of 1∶8,000 and 1∶50,000, respectively. Blots were incubated with polyclonal affinity–purified rabbit antiserum against the carboxy terminus of FGFR4 (Santa Cruz, Santa Cruz, CA). Immunoblotting was performed using anti-FGFR4 (Santa Cruz, 1∶1000), a monoclonal antibody to the V5-tag (Invitrogen, Burlington, ON), anti-MnSOD (Millipore, Billerica, USA), anti-FRS2α (R&D systems, Minneapolis, USA, 1∶1000), anti-Erk1/2 (Sigma, 1∶10000), anti-pFRS2α (Y196, 1∶1000), anti-pErk1/2 (1∶1000), anti-pY-STAT3 (Y705, 1∶1000), pS-STAT3 (S727, 1∶1000), STAT3 (1∶2500), anti-pSrc (Y416, 1∶1000), Src (1∶1000), anti-Tubulin (1∶1000), anti-acetylated Histone H3 (1∶3000) were purchased from Cell Signaling (Pickering, ON). Loading was monitored by detection of actin (1∶500, Sigma). Non-specific binding was blocked with 5% nonfat milk in 1× TBST (Tris-buffered saline with 0.1% Tween-20). After washing for 3×10 mins in 1× TBST, blots were exposed to the secondary antibody (anti-mouse or rabbit IgG-HRP, Santa Cruz) at a dilution of 1∶2000 and were visualized using ECL chemiluminescence detection system (Amersham, U.K.).

### Immunofluorescence detection of phospho-serine STAT3

Cells were grown in 2 chamber slides and pre-incubated in serum-free define medium for 16 hrs. Cells were incubated with MitoTracker Red CMXRos (Invitrogen) at 37°C for 20 minutes, washed twice with PBS, fixed with 4% formaldehyde/PBS for 10 minutes, and washed three times with PBS. Cells were permeabilized for 10 minutes in PBS with 0.2% Triton X-100 and blocked for 30 minutes with PBS containing 5% FBS. Cells were first incubated with rabbit anti-STAT3 antibody (1∶100) or anti- pS-STAT3 antibody (1∶100) for 30 minutes at room temperature, washed three times with PBS, subsequently incubated with anti-rabbit IgG Alexa Fluor 488 for 30 minutes at room temperature, and washed three times with PBS. Coverslips were mounted in Fluoromount-G purchased from Electron Microscopy Sciences (Hatfield, PA) on glass slides. Cells were examined with Two-photon microscope (Zeiss LSM 510 META NLO), equipped with a 63× water-immersion objective lens and filters optimized for double-label experiments. Images were analyzed using the LSM IMAGE browser.

### Fgfr4-R385 knock-in mice

Fgfr4-R385 knock-in (KI) mouse were generated using standard approaches as described previously [28]. Mice were maintained on a pure C57BL/6 background. Genotyping was performed by PCR of genomic tail-DNA [28]. The care of animals was approved by the Institutional Animal Care facilities. Serum IGF-1 (Quantikine ELISA kit) and Prolactin (Calbiotech) levels were measured according to the manufacturer's protocols. Tissues were frozen in liquid nitrogen and stored at −70°C, or fixed in formalin and embedded in paraffin for histologic and immunohistochemical analyses. At least 6 animals were included at each time point for experimental measures.

### Immunocytochemistry

Pituitary glands were stained with the Gordon-Sweet silver method to demonstrate the reticulin fiber network. Immunocytochemical stains to localize adenohypophysial hormones were performed as previously reported [17]. Primary polyclonal antisera directed against rat pituitary hormones were used at the specific dilutions: GH, 1∶2500; prolactin, 1∶2500; ß-thyroid-stimulating hormone (ß-TSH), 1∶3000; ß-follicle-stimulating hormone (ß-FSH), 1∶600; ß-luteinizing hormone (ß-LH), 1∶2500 (National Hormone and Pituitary Program, Rockville, MD); and adrenocorticotropin pre-diluted preparation, which was further diluted 1∶20 (Dako, Carpinteria, CA).

### Human samples

Human pituitary tissues were retrieved from the files of the University Health Network with REB approval. All had been fully characterized according to currently accepted criteria [45]. Fasting serum growth hormone levels were obtained from patients with histologically proven GH-producing pituitary adenomas. Pituitary tumor size was based on the maximal diameter noted on magnetic resonance imaging (MRI). FGFR4 germline genotyping was performed on DNA isolated from circulating while blood cells as described [21].

### Ethics statement

The care of animals was approved by the Institutional Animal Care facilities. Human pituitary tumors were retrieved from the files of the University Health Network with REB approval.

### Statistical analysis

Data are presented as mean ± standard deviation (SD). In the experimental models, differences were assessed by the unpaired, two-sided *t* test. *P*<0.05 was considered statistically significant. The analysis of surgical human tumor specimens applied Fisher's exact test.

## Supporting Information

Figure S1 Endogenous Fgfr4 expression in pituitary cell lines and primary pituitaries of Fgfr4-R385 knock-in mice. Fgfr4 protein expression was examined by western blotting in the indicated GH4 and PRL235 cell lines and in primary pituitaries of Fgfr4-R385 knock-in mice. Native HEK239 cells show minimal FGFR4 reactivity.
(TIF)

Figure S2 STAT3 serine phosphorylation induces pituitary tumor cell growth. GH4 cells expressing wild-type (STAT3-WT), inactive (STAT3-Y705F), constitutively active (STAT3-S727D), or inactive (STAT3-S727A) STAT3 were grown in soft agar. Shown are percentages compared to vector controls and expressed as a mean ± SD of measurements obtained from four independent experiments. Statistically significant differences are depicted as *p<0.05 or **p<0.01.
(TIF)

Figure S3 STAT3 down-regulation alters the Growth hormone/Prolactin balance in GH4 cells. GH4 cells expressing FGFR4-G388 were transfected with siRNA oligonucleotides directed at STAT3. This forced STAT3 reduction results in increased GH and reduced PRL as shown by western blotting with corresponding densitometric values immediately below.
(TIF)
