# Supplementary figures and images for: The FGFR4-G388R Polymorphism Promotes Mitochondrial STAT3 Serine Phosphorylation to Facilitate Pituitary Growth Hormone Cell Tumorigenesis
Source: PLoS Genet. 2011 Dec 8;7(12):e1002400. doi: 10.1371/journal.pgen.1002400 (PMC3234213; doi:10.1371/journal.pgen.1002400)

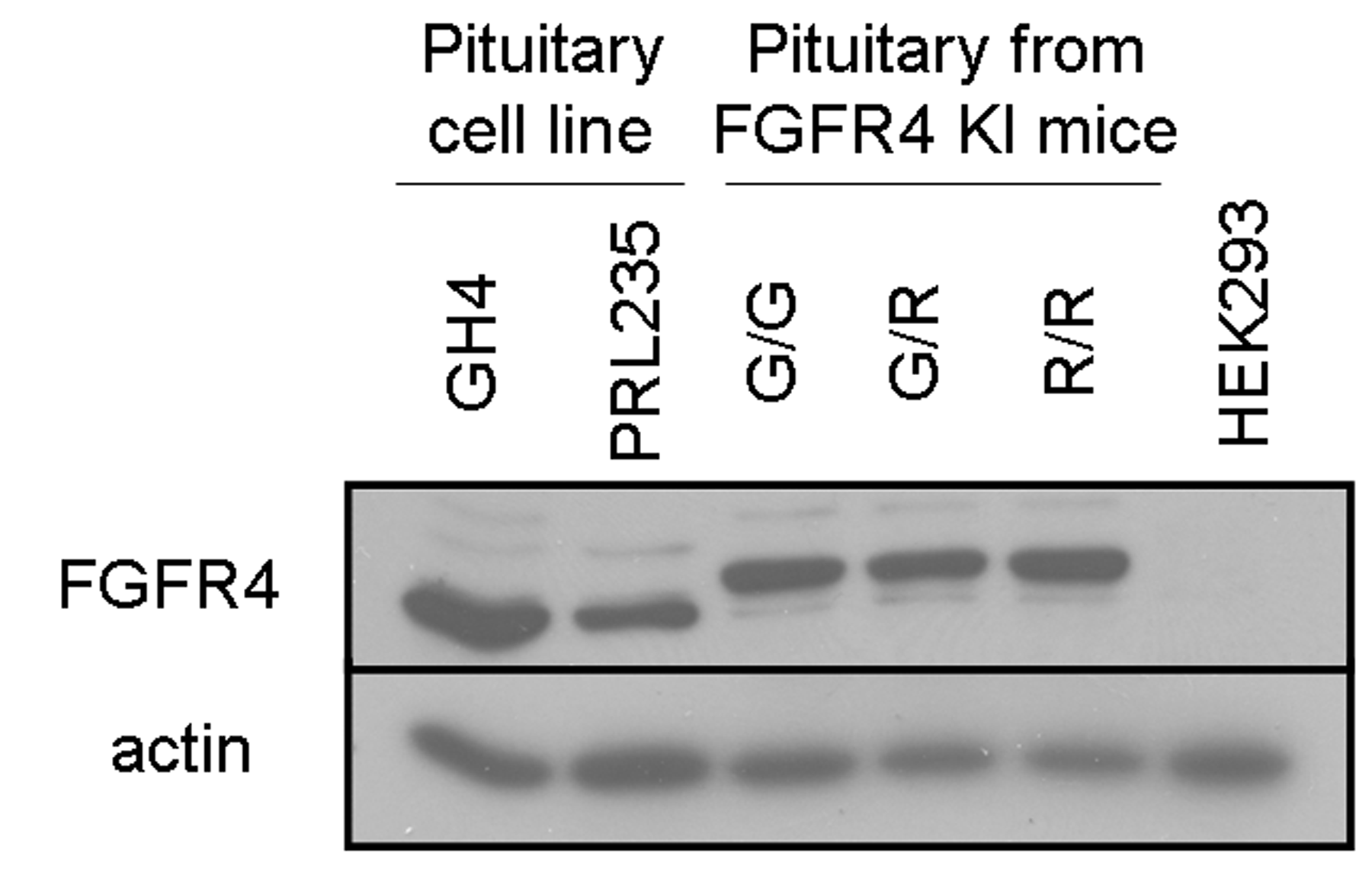

Supplement: Figure S1 — Endogenous Fgfr4 expression in pituitary cell lines and primary pituitaries of Fgfr4-R385 knock-in mice. Fgfr4 protein expression was examined by western blotting in the indicated GH4 and PRL235 cell lines and in primary pituitaries of Fgfr4-R385 knock-in mice. Native HEK239 cells show minimal FGFR4 reactivity. (TIF) [file pgen.1002400.s001.tif]

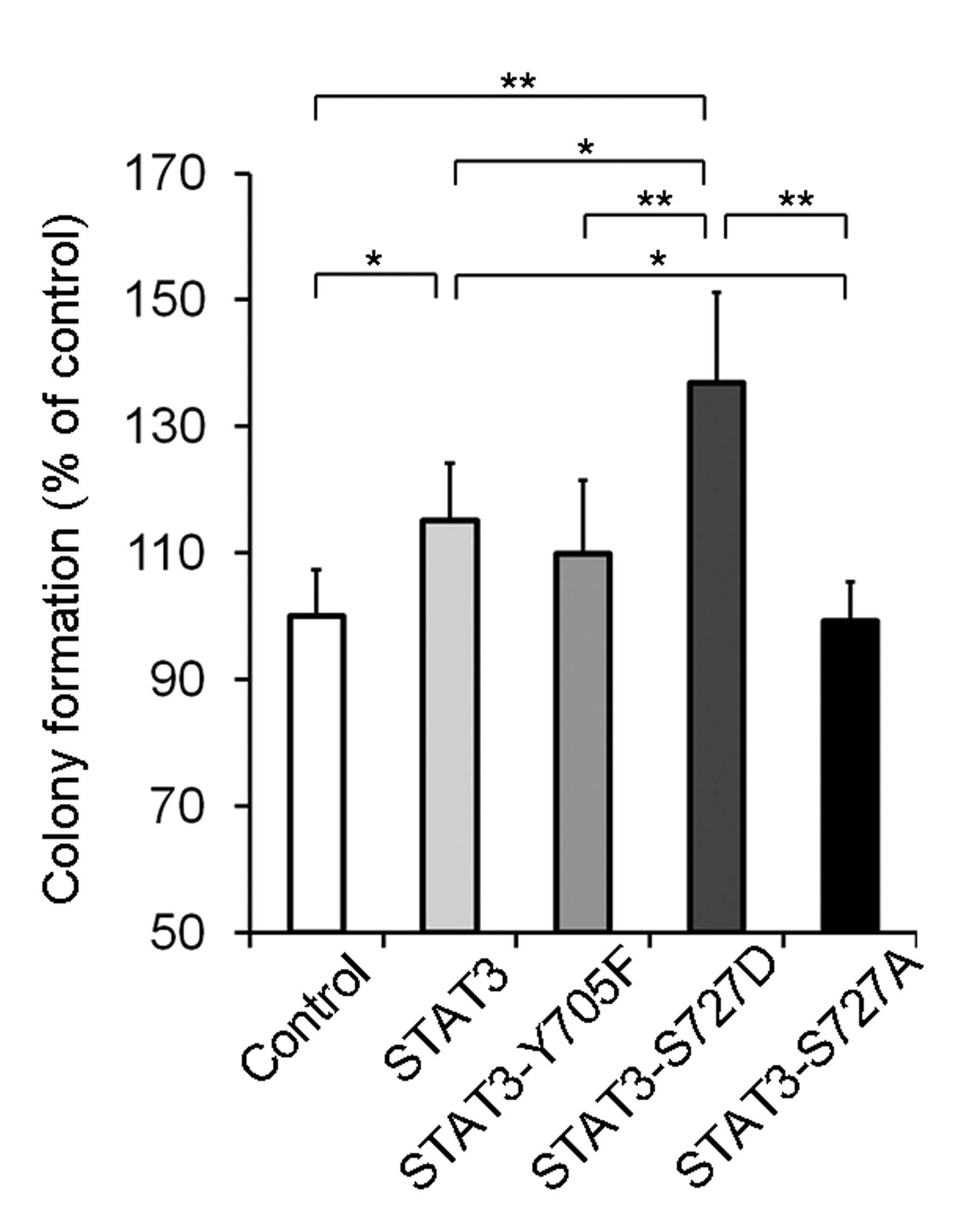

Supplement: Figure S2 — STAT3 serine phosphorylation induces pituitary tumor cell growth. GH4 cells expressing wild-type (STAT3-WT), inactive (STAT3-Y705F), constitutively active (STAT3-S727D), or inactive (STAT3-S727A) STAT3 were grown in soft agar. Shown are percentages compared to vector controls and expressed as a mean ± SD of measurements obtained from four independent experiments. Statistically significant differences are depicted as *p<0.05 or **p<0.01. (TIF) [file pgen.1002400.s002.tif]

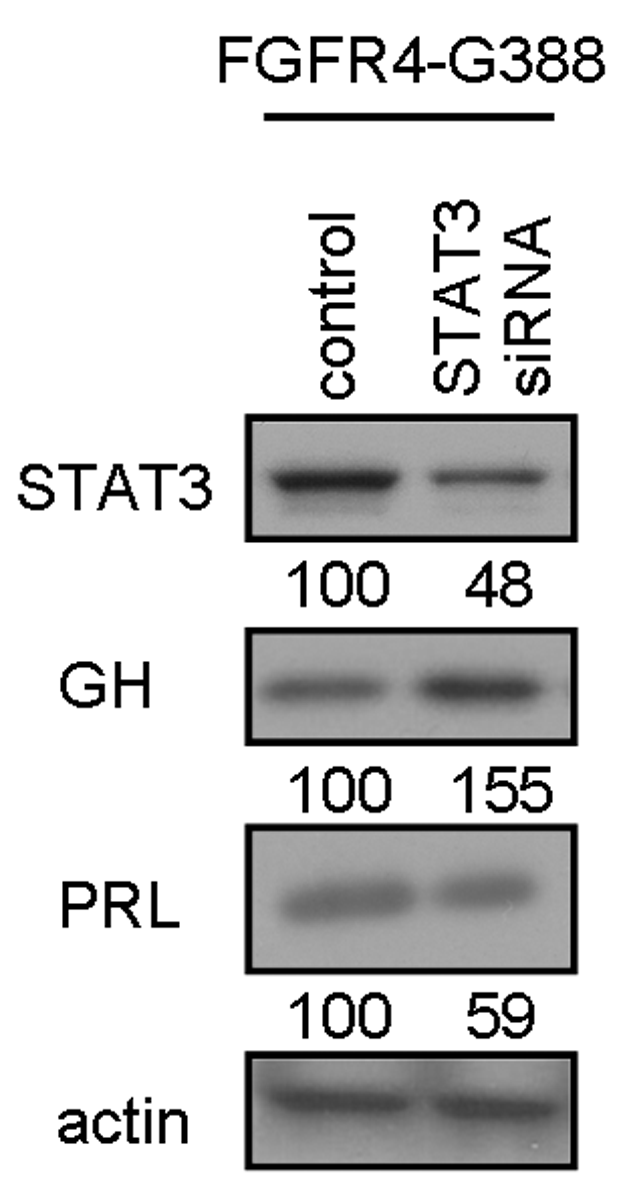

Supplement: Figure S3 — STAT3 down-regulation alters the Growth hormone/Prolactin balance in GH4 cells. GH4 cells expressing FGFR4-G388 were transfected with siRNA oligonucleotides directed at STAT3. This forced STAT3 reduction results in increased GH and reduced PRL as shown by western blotting with corresponding densitometric values immediately below. (TIF) [file pgen.1002400.s003.tif]
